# A Novel Method Ensuring an Immediate Target Angle After Horizontal Strabismus Surgery in Children

**DOI:** 10.3389/fmed.2022.791068

**Published:** 2022-02-24

**Authors:** Joa-Jing Fu, Meng-Wei Hsieh, Lung-Chi Lee, Po-Liang Chen, Liang-Yen Wen, Yi-Hao Chen, Ke-Hung Chien

**Affiliations:** ^1^Dr. Fu Eye Clinic, Taipei, Taiwan; ^2^Department of Ophthalmology, Taoyuan Armed Forces General Hospital, Taoyuan, Taiwan; ^3^Department of Ophthalmology, Tri-Service General Hospital and National Defense Medical Center, Taipei, Taiwan; ^4^Hau-Ming Eye Clinic Center, New Taipei City, Taiwan

**Keywords:** pediatric ophthalmology, strabismus, immediate target angle, horizontal strabismus operation, horizontal strabismus surgery

## Abstract

**Purpose:**

Pediatric strabismus surgery has low success rates and high reoperation rates because of difficult alignment measurements and the nature of different strabismus types. Furthermore, adjustable sutures are not easily employed in children on an OPD basis.

**Methods:**

This was a retrospective comparative case study of children less than 12 years old who underwent strabismus surgery and were followed up at least 6 months postoperatively. We proposed a novel method that combines adjustable sutures and corneal light reflexes in regular strabismus surgery to improve surgical results. Efficacy and safety were evaluated and compared with those in a regular fixed-suture group.

**Results:**

In total, 128 children (88: exotropia and 41: esotropia) in the novel method group (Group 1) and 109 (71: exotropia and 38: esotropia) in the regular fixed-suture group (Group 2) were enrolled. The primary outcome was the immediate target angle (for esotropia within 4 PD of orthotropia and exotropia within 8 PD of esotropia within the first week postoperatively); the secondary outcome was success at the 6-month visit (angle of deviation < 10 PD). Consequently, there was a significantly higher proportion of achieving the immediate target range and success rate in both exotropic and esotropic patients in Group 1 than in Group 2. A significantly lower reoperation rate was also demonstrated in Group 1. No complications were noted in either group.

**Conclusions:** The novel method enabled a higher proportion of subjects to achieve an immediate target range and success rate and a lower chance of reoperation among both esotropic and exotropic patients.

## Introduction

Strabismus surgery is a common procedure, but it is usually difficult in children due to assess precise angle of deviation before operation. Owing to the difficult measurement of strabismus in children (1) and the nature of different types of strabismus, there is a higher reoperation rate among children than among adults (2–5). Older age of children can ensure a better performance in strabismus measurements, but children who are operated on at an older age have a higher rate of reoperation (6). It seems essential to pursue surgery performed within the critical period as well as higher prediction of surgical results to reduce the reoperation rate.

To improve the prediction of postoperative alignment, the adjustable suture technique is a common and reliable procedure in strabismus surgeries to improve the success rate (7–9). However, compared to most adjustments that could be made in the office only by local anesthetics, adjustments in children seem to be more challenging and painful for both the patients and their parents despite some alternatives in anesthetics (9). To improve the success rate in pediatric strabismus surgery, certain alternative procedures are combined with strabismus surgeries, such as botulinum toxin A injection (10, 11) and pull-string adjustments (12). However, a need for additional procedures cannot be avoided (13). Therefore, the combination of procedures in the same session with strabismus surgery seems to be a better approach in this issue.

Recently, postoperative measurements of the angle of deviation within 1 week, the “target angle” or “immediate target angle”, have seemed to be predictors correlated with long-term surgical success in both adults and children (14, 15). Therefore, a procedure to ensure a higher rate of patients meeting the criteria of an immediate target angle in children is currently unmet. In this study, we intend to propose a novel method to improve the success rate of horizontal strabismus surgeries in children. Additionally, the efficacy of this method in achieving an immediate target angle as the primary outcome and meeting success criteria at 6 months after strabismus surgery as the second outcome were also evaluated.

## Methods

This retrospective study was conducted to collate data on strabismic children who underwent strabismus correction surgeries in Tri-Service General Hospital or Dr. Fu Eye Clinic from January 2011 to December 2020. The study protocol and supporting documents were reviewed and approved by the institutional review board (No: C202105113) of the Tri-Service General Hospital, Taipei, Taiwan. The requirement for informed consent was waived by the review board due to the retrospective basis of the study. All of the patients who were chosen for the study had visited our ophthalmology department or Dr. Fu Eye Clinic for regular follow-ups for at least 6 months after strabismus surgery.

Patients were eligible for inclusion in the study if they were aged younger than 12 years at the initial visit and the time of strabismus operation at either one of two centers and had no comorbidity of other ocular diseases, such as uncorrected amblyopia (defined as a reduction in best corrected visual acuity (BCVA) without an attributable structural anomaly); furthermore, amblyopia due to any cause had to be corrected prior to their strabismus surgery. Patients were excluded from the study if they had other concurrent ocular diseases that might result in uncorrectable amblyopia or if they had follow-up period of less than 6 months after their operation.

**Figure 1:**
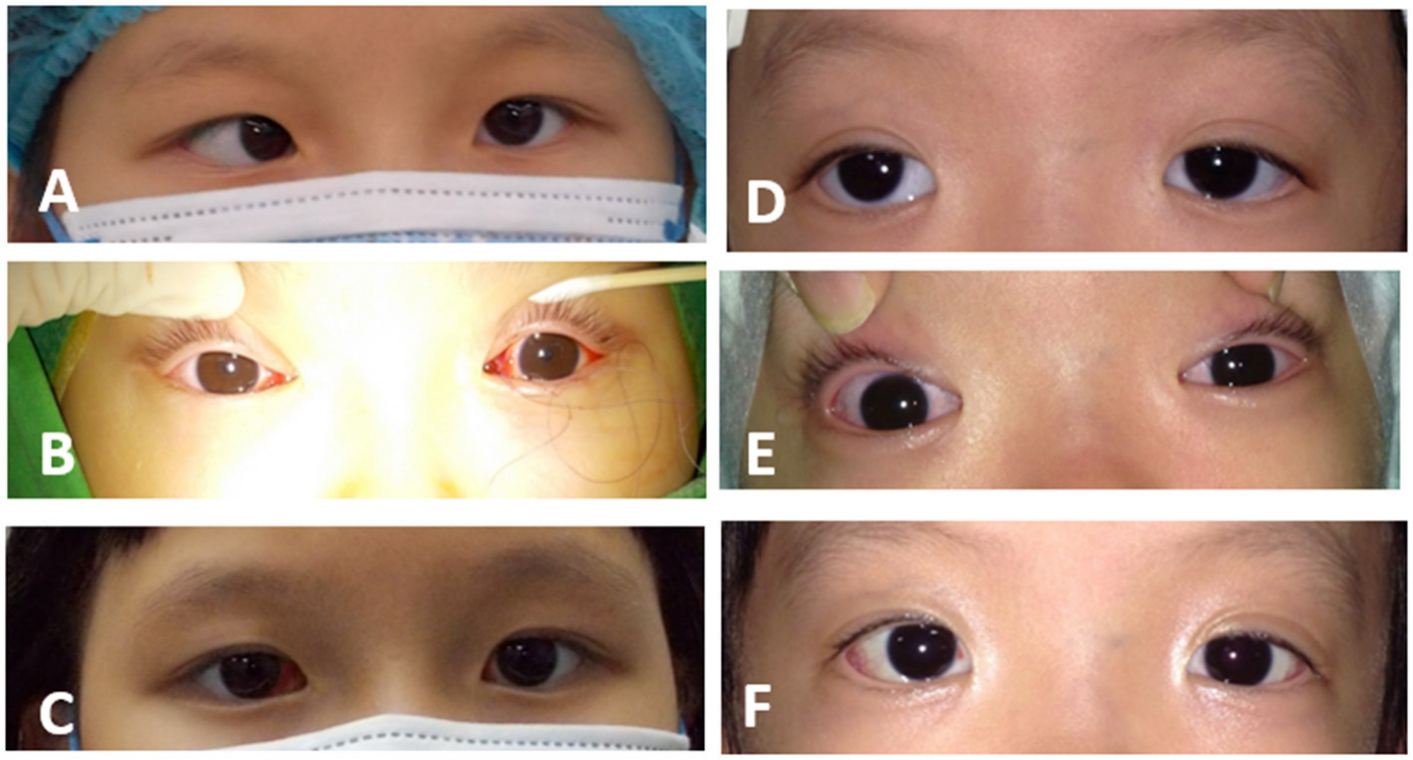
Perioperative photographs of patients who underwent the novel method. **(A)** A 6-year-old girl showed 45 PD esotropia before the operation. **(B)** Adjustment was performed intraoperatively with assistance of the corneal light reflex. **(C)** At the 1-week postoperative visit, the girl showed orthophoric results. **(D)** A 5-year-old girl presented with 45 PD exotropia before the operation. **(E)** The end point of adjustment was shown during strabismus surgery. **(F)** The girl showed orthophoria at her 1-week postoperative visit.

The surgical technique performed at Dr. Fu Eye Clinic was a modification of the adjustable suture technique performed at the end of routine strabismus surgery. Briefly, the conjunctival wound was created through a forniceal approach in either the inferomedial or inferolateral quadrant. Recession, resection and a combination of these two procedures were performed on a routine basis with 6-0 Vicryl and fixed on the sclera, but a slipknot of the scleral suture was temporarily left. Light spots reflected on the cornea of both eyes were checked with a loupe light. The temporary slipknot was then adjusted until the light spot of one eye stayed at the position of 1.5 mm nasal to the pupil center while the other light spot stayed in the center of the pupil (Figure 1). The slipknot was then tied to a dead knot. Additional surgery was performed on a traditional basis, such as inferior oblique muscle (IO)-weakening procedures in IO overaction (IOOA), correction of epiblepharon, and frontalis sling procedures for congenital ptosis. Finally, the conjunctival wound was closed with 8-0 Vicryl.

The surgical technique performed at Tri-Service General Hospital was routine. Recession, resection and combination surgery were performed with a forniceal approach and scleral fixation with 6-0 Vicryl. Surgical doses were determined by the surgeon's preference, but no adjustment was made in the surgery. Other procedures, including additional surgeries and conjunctival wound closure, were performed in the same manner.

The following parameters were collected from the charts for the study: age at the time of study, sex, BCVA, history of amblyopia or other comorbidities (such as congenital ptosis, epiblepharon, development delay, and prematurity), binocularity by stereopsis, type and measurement of strabismus, surgical type of strabismus, surgical complications, history of a strabismus operation, and alignment within the first postoperative week at the 1-month, 3-month, and 6-month follow-up visits. Complications and reoperations, if any, were also collected during the following period.

Binocularity was defined as achieving better than 100 s of arc on the Titmus circles test with the angle of strabismus corrected with prisms (16). Strabismus measurements were performed with the alternate prism and cover test (APCT) in most children and the modified Kirmsky test in children who were too young or uncooperative to perform the alternative prism cover test (APCT), such as those with developmental delays. Surgical planning was performed after at least two reliable measurements recorded within the 5 prism diopter (PD) difference.

Outcome measures were collected from the charts as (1) the primary outcome: the angle of deviation measured at the postoperative week in the “immediate target angle” and classified as within the target range or not. The target range was referenced from Astudillo et al. (15) for esotropia that was within 4 PD of orthotropia and for exotropia that was within 8 PD of esotropia (2). The secondary outcome was success of surgery as modified from Astudillo et al. (15) and was defined as an angle of deviation less than 10 PD at the 6-month follow-up visit.

The data were analyzed using SPSS software version 18.0 for Windows (SPSS Inc., Chicago, IL, USA). Non-normal distribution was confirmed with the kolmogorov-smirnov normality test. And then non-parametric statistics were applied in the study. The Chi-Square Test was applied in the difference of categorical variables between two groups while the Fisher Exact Test was applied in certain variables. The Mann-Whitney *U* test was applied in the difference of continuous variables between two groups. A *P* < 0.05 was considered statistically significant.

## Results

A total of 128 subjects underwent the novel method during their strabismus surgery at Dr. Fu Eye Clinic (Group 1), and 109 subjects underwent traditional strabismus surgery without adjustment sutures from Tri-Service General Hospital (Group 2), who met the study criteria and underwent strabismus surgery for either esotropia or exotropia between January 2011 and December 2020. In Group 1, there were 74 male patients (57.8%) and 54 female patients (42.2%), with a mean age (SD) of 6.24 (2.78) years, and a mean (SD) follow-up period of 14.67 (7.81) months. In Group 2, there were 69 male patients (63.3%) and 40 female patients (36.7%), with a mean age (SD) of 7.36 (3.66) years, and a mean (SD) follow-up period of 16.44 (8.23) months. None of the baseline characteristics (except sex) significantly differed between the groups (Table 1).

**Table 1:** Demographic characteristics of children in the study.

	**Group 1**	**Group 2**	
No. of subjects (*N*)	128	109	
Male (*N*) (%)	74 (57.8)	69 (63.3)	*P* = 0.04^*^
Female (*N*) (%)	54 (42.2)	40 (36.7)	
Baseline age (years) (mean) (SD)	6.24 (2.78)	7.36 (3.66)	*P* = 0.63
Baseline age (years) (min) (max)	(1.1) (11.9)	(1.3) (11.9)	
Follow-up periods (month) (mean) (SD)	14.67 (7.81)	16.44 (8.23)	*P* = 1.02
Follow-up periods (min) (max)	(6.3) (60.0)	(6.1) (38.6)	
Binocularity (*N*) (%)	82 (64.1)	62 (56.9)	*P* = 0.86
Prematurity (*N*) (%)	6 (4.7)	11 (10.1)	*P* = 0.02^*^
Development delay (*N*) (%)	1 (0.8)	2 (1.8)	*P* = 1.33
Duane type 1 (*N*) (%)	1 (0.8)	0	*P* = 1.47
Optic pit (*N*) (%)	1 (0.8)	0	*P* = 1.47
Corrected amblyopia (*N*) (%)	13 (10.2)	8 (7.3)	*P* = 0.81
Prior strabismus surgery (*N*) (%)	16 (12.5)	23 (21.1)	*P* < 0.01^*^
Exotropia (*N*) (%)	88 (68.8)	71 (65.1)	*P* = 0.66
Esotropia (*N*) (%)	40 (31.2)	38 (34.9)	
**Additional operation**			
Ptosis correction (*N*) (%)	1 (0.8)	0	*P* = 1.47
Epiblepharon correction (*N)* (%)	1 (0.8)	6 (5.5)	*P* < 0.01^*^
IOOA correction (*N*) (%)	18 (14.1)	13 (11.9)	*P* = 0.71
Other vertical muscle surgery (*N*) (%)	1 (0.8)	0	*P* = 1.47

*The P value in the table is from values compared between groups. SD, standard deviation. IOOA, inferior oblique muscle overaction*.

* *Significance*.

Tracing the ophthalmic status before the operation, 82 patients (64.1%) in Group 1 and 62 patients (56.9%) in Group 2 achieved binocularity before the operation, with a non-significant difference. In the underlying history, only the parameters of prematurity (6/128, 4.69 in Group 1 and 11/109, 10.09% in Group 2, *p* = 0.02) and history of prior surgery (16/128, 12.5 in Group 1 and 23/109, 21.10% in Group 2, *p* < 0.01) were significantly different between the groups (Table 1).

To study the novel method regarding treatment success in these patients, we evaluated the type of strabismus.

In patients with exotropia, there were 42 male patients (47.7%) and 46 female patients (52.3%) in Group 1 and 36 male patients (50.7%) and 35 female patients (49.3%) in Group 2 (*P* = 0.812). In the age distribution, there was a mean of 6.40 years (SD 2.68) in Group 1 and 7.81 years (SD 3.13) in Group 2, and the difference was significant (*P* = 0.03). Regarding the surgery type, the major operation in both groups was bilateral lateral rectus muscle recession (80/88 in Group 1, 90.9 and 66/71 in Group 2, 92.9%, *P* = 0.112). The mean angle of deviation before surgery was 37.64 PD (SD 8.1) in Group 1 and 35.73 PD (SD 10.22) in Group 2 (*P* = 0.16). After the operation, the most important indicator was the angle of deviation within the first week (the immediate angle). The immediate angle was reported to be associated with surgical success in children (15). In our study, the mean angle of deviation in the first postoperative week was 1.02 PD esotropia (SD 4.51) in Group 1 and 6.5 PD esotropia (SD 7.55) in Group 2, which showed a significant difference (*P* < 0.01). There were 75 patients in Group 1 (85.2%) and 47 patients in Group 2 (66.2%) (*P* < 0.01) who met the criteria of the immediate target range. We collected charts of patients who had been followed up for at least 6 months after their operation, and the angle of deviation at the 6-month postoperative visit was used to judge the surgical result as success or failure because it is reported that recurrence mostly occurs 6 months after the surgery (17). As a result, in our study, the mean angle of deviation at the 6-month postoperative visit was 0.64 PD exotropia (SD 3.65) in Group 1 and 5.75 PD esotropia (SD 17.93) in Group 2, which was significantly different (*P* < 0.01). There were 85 patients (95.5%) in Group 1 and 52 patients (73.2%) in Group 2 with significant intergroup differences (*P* < 0.01) (Table 2).

**Table 2:** Detailed information on exotropic patients.

	**Group 1**	**Group 2**	
No. of subjects (*N*)	88	71	
Male (*N*) (%)	42 (47.7)	36 (50.7)	*P* = 0.812
Female (*N*) (%)	46 (52.3)	35 (49.3)	
Baseline age (years) (mean) (SD)	6.4 (2.68)	7.81 (3.13)	*P* = 0.03^*^
Baseline age (years) (min) (max)	(2.8) (11.9)	(3.5) (11.9)	
**Surgery type**			
Single muscle recession (*N*) (%)	6 (6.8)	3 (4.2)	*P* = 0.06
Recession and resection (*N*) (%)	2 (2.3)	2 (2.8)	*P* = 1.52
Bilateral muscles recession (*N*) (%)	80 (90.9)	66 (92.9)	*P* = 0.11
Preoperative angle of deviation (PD) (mean) (SD)	37.64 (8.1)	35.73 (10.2)	*P* = 0.16
**Postoperative angle of deviation (PD) (mean) (SD)**			
One week	−1.02 (4.51)	−6.5 (7.55)	*P* < 0.01^*^
One month	0.65 (9.19)	−4.67 (8.08)	*P* < 0.01^*^
Three months	1.07 (3.75)	−1.01 (9.90)	*P* = 0.03^*^
Six months	0.64 (3.65)	−5.75 (10.42)	*P* < 0.01^*^
Final visit	1.05 (6.12)	−8.67 (17.93)	*P* < 0.01^*^
Within the immediate target range (N) (%)	75 (85.2)	47 (66.2)	*P* < 0.01^*^
Success (N) (%)	85 (95.5)	52 (73.2)	*P* < 0.01^*^
Reoperation (N) (%)	4 (4.5)	7 (9.9)	*P* < 0.01^*^

*The P-value in the table is from values compared between groups. SD, standard deviation. PD, prism diopter. A negative PD value indicates esotropia, and a positive PD value indicates exotropia*.

* *Significance*.

Among the patients with esotropia, there were 32 male patients (80.0%) and 8 female patients (20.0%) in Group 1 and 33 male patients (86.8%) and 5 female patients (13.2%) in Group 2 (*P* = 0.132). In the age distribution, there was a mean age of 5.86 years (SD 3.02) in Group 1 and a mean age of 5.32 years (SD 3.43) in Group 2 (*P* = 0.06). Regarding the surgery type, the major operation in both groups was bilateral medial rectus muscle recession (37/40 in Group 1, 92.5; 36/38 in Group 2, 94.7%; *P* = 0.201). The mean angle of deviation before surgery was 30.21 PD (SD 17.91) in Group 1 and 32.36 PD (SD 14.65) in Group 2 (*P* = 0.06). The mean angle of deviation in the first postoperative week was 0.85 PD esotropia (SD 4.06) in Group 1 and 2.68 PD esotropia (SD 3.56) in Group 2, which was significantly different (*P* = 0.02). There were 32 patients in Group 1 (80.0%) and 25 patients in Group 2 (65.8%) (*P* < 0.01) who met the criteria of the immediate target range. In the evaluation of surgical success on the exam at the 6-month postoperative visit, the mean angle of deviation was 0.70 PD esotropia (SD 5.35) in Group 1 and 6.78 PD esotropia (SD 5.12) in Group 2, which was significantly different (*P* < 0.01). As a result, there were 38 patients (95.0%) in Group 1 and 27 patients (71.1%) in Group 2, with significant intergroup differences (*P* < 0.01) (Table 3).

**Table 3:** Detailed information on esotropic patients.

	**Group 1**	**Group 2**	
No. of subjects (*N*)	40	38	
Male (*N*) (%)	32 (80.0)	33 (86.8)	*P*= 0.132
Female (*N*) (%)	8 (20.0)	5 (13.2)	
Baseline age (years) (mean) (SD)	5.86 (3.02)	5.32 (3.43)	*P*= 0.06
Baseline age (years) (min) (max)	(1.1) (11.9)	(1.3) (9.6)	
**Surgery type**			
Single muscle recession (*N*) (%)	3 (7.5)	2 (5.3)	*P* = 0.13
Bilateral muscle recession (*N*) (%)	37 (92.5)	36 (94.7)	*P* = 0.20
Preoperative angle of deviation (PD) (mean) (SD)	−30.21 (17.91)	−32.36 (14.65)	*P* = 0.06
**Postoperative angle of deviation (PD) (mean) (SD)**			
One week	−0.85 (4.06)	−2.68 (3.56)	*P* = 0.02^*^
One month	−1.73 (4.77)	−3.79 (4.84)	*P* = 0.02^*^
Three months	−0.96 (4.12)	−4.12 (4.21)	*P* < 0.01^*^
Six months	−0.70 (5.35)	−6.78 (3.87)	*P* < 0.01^*^
Final visit	−0.39 (5.68)	−6.86 (5.12)	*P* < 0.01^*^
Within the immediate target range (*N*) (%)	32 (80.0)	25 (65.8)	*P* < 0.01^*^
Success (*N*) (%)	38 (95.0)	27 (71.1)	*P* < 0.01^*^
Reoperation (*N*) (%)	1 (2.5)	3 (7.9)	*P* < 0.01^*^

*The P-value in the table is from values compared between groups. SD, standard deviation. PD, prism diopter. A negative PD value indicates esotropia, and a positive PD value indicates exotropia*.

* *Significance*.

The novel method resulted in a significantly higher rate in Group 1 than that of the traditional scleral-fixation method in Group 2, in both the parameter of achieving the immediate target angle (83.6% in Group 1 and 66.1% in Group 2, *P* < 0.01) and that of the success rate at the 6-month postoperative visit (96.1% in Group 1 and 72.5% in Group 2) (Table 4).

**Table 4:** Primary and secondary outcomes in each group.

	**Group 1**	**Group 2**	
No. of subjects (*N*)	128	109	
Within the immediate target range (*N*) (%)	107 (83.6)	72 (66.1)	*P* < 0.01^*^
Success (*N*) (%)	123 (96.1)	79 (72.4)	*P* < 0.01^*^

*The P-value in the table is from values compared between groups*.

* *Significance*.

In the evaluation of reoperation in exotropic patients, there were 4 patients in Group 1 (4/88, 4.50%) and 7 patients in Group 2 (7/71, 9.86%) who underwent reoperation during the subsequent periods (*P* < 0.01). Similar to that for esotropia, the reoperation rate was 2.50% in Group 1 (1/40) and 7.89% in Group 2 (3/38). No complications, such as slipped muscles, wound infection, granulomas or vision loss after the operations, were recorded in either group.

## Discussion

In this study, we proposed a novel method in children with a higher rate of achieving an immediate target angle (83.6 in Group 1 and 66.1% in Group 2) and success rate at the 6-month postoperative visit (96.1 in Group 1 and 72.5% in Group 2). Briefly, the method was designed based on two prior methods with modifications to facilitate strabismus measurement and surgical precision in children (14, 18, 19). Based upon the observation of light spots in patients with orthophoria who underwent other kinds of eye surgeries under general anesthesia, most of their eyes exhibited a light spot ~1 to 2 mm nasal to the pupil center in one eye while in the center of the other eye during a corneal light reflex exam conducted with a coaxial loupe light under general anesthesia; we applied this event as a surgical goal (1.5 mm nasal to the pupil center) in the adjustment during regular strabismus surgery. The results revealed that this method could provide both an effective and safe approach in strabismus surgery in children.

Since it was introduced in 1977 (20), adjustable suture in strabismus surgery has been viewed as a safe and effective procedure to improve surgical results. Zhang et al. (7) concluded their results in adults, and adjustable sutures significantly raised the surgical success rate from 61.3 in the nonadjustable group to 74.8% in the adjustable group, especially concerning the reoperation status. However, the latest studies showed no significant difference in adult surgery, with a success rate of 61.7 in the adjustable group and 60.3% in the non-adjustable group at the 1- to 2-month postoperative visits (21). Some studies have stated that adjustable sutures are also safe and effective in pediatric strabismus surgery. Chan et al. (8) reported their results in 37 esotropic and 52 exotropic children between 7 and 15 years of age, where 25 patients (25/89, 28.1%) were adjusted after strabismus correction and achieved a 74.2% success rate (only one patient experienced slipped-muscle complications). Later, in 2008, Awadein et al. (9) reported their experience in children younger than 10 years and found similar results in terms of a higher success rate. They also proposed that adjustable sutures were more effective for esotropias. However, these results are not supported by the only randomized controlled trials from the literature in which Kamal et al. (22) reported a non-significant difference in success rate but a trend higher in the adjustable suture group (86.67 vs. 73.33%, *P* = 0.197) among children less than 12 years old. Clearly, adjustable sutures help strabismus patients, but they cannot be performed under local anesthesia in children (12). Our novel method combined adjustable sutures in the same session as regular strabismus surgery and therefore reduced the need for adjustment.

The definition of success in strabismus surgery varies among different studies. Most studies set their success criteria as 8 to 10 PD deviations from orthophoria at either the 3-month, 6-month or last follow-up (8, 9, 12, 15, 21–24). Kushner et al. (25) found that orthotropia in infants at their 6-month postoperative visits was more related to better visual function outcomes 5 years later. It is clear that postoperative drift in strabismus surgery must be considered a surgical progress in defining surgical success. Herein, we only collected patients with a minimum postoperative visit at 6 months to better evaluate the efficacy of the novel method, although some patients may be excluded due to failure to meet the follow-up criteria even if they had good surgical results.

In the consideration of postoperative drift in strabismus surgeries, Leow et al. (26) found that there was a good correlation of postoperative results between the 1-week and 6-month visits. Choi et al. (27) also found that mild overcorrection at 1 week postoperatively was correlated with low recurrence of exotropia within 2 years. This implies the importance of ocular alignment at the first postoperative week. In 2015, the term “immediate target angle” was proposed as 0–8 PD esotropia in exotropic patients and within 4 PD of orthophoria in esotropic patients at a follow-up visit within the first week after the surgery (14, 15). Mireskandari et al. (14, 15) stated that patients who achieved an immediate target angle after strabismus surgery were more likely to have long-term success in exotropic adults and children. Adjustable sutures could help even more patients reach the target angle after adjustment. We also followed the setting and measured all of our patients during the first week postoperatively in daily practice. In our study, there was a significantly higher proportion of patients who met the target angle in Group 1 than in Group 2 (85.2 vs. 66.2%, *P* < 0.01). Consistent with prior studies, cases within the target angle were significantly related to success (104/107, 97.2 in Group 1; 64/72, 88.9% in Group 2). Prior study results showed that exotropic patients were more prone to success if they met the immediate target rate than esotropia patients were (14, 15). In contrast to their results, our study did not find a difference in the target angle related to the success rate between esotropic and exotropic patients in either group (*P* = 0.262). However, patients in Group 1 demonstrated a significantly higher proportion of achieving the target angle and then a higher success rate in both esotropic and exotropic patients.

The type of strabismus influences its characteristics in different aspects, such as the extent and time-course of postoperative drift (14, 15, 27–32), response to adjustable sutures (9), recurrence (17), and success rate (23, 33). In exotropia, it is noted to have a larger and longer postoperative drift (30). Park et al. (34) reported their experience with a median survival time of esodrifts as long as 2.0 ± 0.1 months in patients older than 15 years, and 6.1% of patients in the group younger than 15 years old even had an esodrift noted at the 6-month postoperative visit. In esotropia, postoperative drift has a relatively minor effect. Wang et al. (24) reported that the deviation changes at postoperative days 1–3 were not significantly different from those at the final follow-up. Pukrushpan et al. (28) found no drift in their esotropic patients and a mean PD deviation of 11.3 (8.6) in exotropic patients. From these observations, there were different target ranges for exotropia and esotropia. Similar progress of postoperative drift was seen in our study: exotropic patients demonstrated drifts, but esotropic patients showed fewer drifts within the 6-month follow-up.

In analyzing the factors leading to undercorrection of patient with intermittent exotropia, Koo et al. stated that dissociated vertical deviation, amblyopia, anisometropia and vertical strabismus had no significant role, while the initial postoperative alignment was the most important factor (33). Astudillo et al. reported that prematurity is related to poor outcome (15). In our study, none of the factors, such as prematurity, corrected amblyopia, prior history of strabismus surgery, or IOOA status, were related to surgical success in either group. We believe that this could have resulted from insufficient cases in these situations. A well-designed trial should be designed to answer these questions.

Our current study still has limitations. First, it was designed to compare subjects from two medical centers on a retrospective basis. Due to a strict inclusion criterion, only patients with regular follow-up for at least 6 months were included in the study. Therefore, some information on subjects who underwent similar surgeries but did not meet the criteria may be lost or hidden. Second, all surgeries collected in this study were performed by two surgeons (Joa-Jing Fu, MD and Ke-Hung Chien, MD). Therefore, selection bias cannot be ignored, but a randomized controlled trial (RCT) would shed light on this issue.

In conclusion, we propose a novel method to combine adjustable sutures in regular strabismus surgery sessions by using the corneal reflex method. A higher proportion of patients achieving the immediate target range and success rate and a lower chance of reoperation among both esotropic and exotropic subjects was shown though a comparative control of regular fixed-suture strabismus surgery. We believe our method could be a treatment choice for strabismus surgeries in children.

## Data Availability Statement

The original contributions presented in the study are included in the article/supplementary materials, further inquiries can be directed to the corresponding author/s.

## Ethics Statement

The study protocol and supporting documents were reviewed and approved by the Institutional Review Board (No: C202105113) of the Tri-Service General Hospital, Taipei, Taiwan. Written informed consent from the participants' legal guardian/next of kin was not required to participate in this study in accordance with the national legislation and the institutional requirements. Written informed consent was obtained from the minor(s)' legal guardian/next of kin for the publication of any potentially identifiable images or data included in this article.

## Author Contributions

P-LC and L-YW: design of the study. J-JF and M-WH: conduct of the study. J-JF and Y-HC: data collection. L-CL: data analysis and preparation. L-CL and K-HC: interpretation of the data. K-HC and P-LC: review. K-HC: approval of the manuscript. All authors contributed to the article and approved the submitted version.

## Funding

This research was funded by the TSGH (Tri-Service General Hospital) (TSGH-E-109231 and TSGH-D-110115), the Taoyuan Armed Forces General Hospital (TYAFGH-E-111044 and TYAFGH-A-110019), the Taiwan Ministry of Science and Technology (MOST 110-2314-B-016-051), and the Ministry of National Defense Medical Affairs Bureau (MND-MAB-C05-111019).

## Conflict of Interest

The authors declare that the research was conducted in the absence of any commercial or financial relationships that could be construed as a potential conflict of interest.

## Publisher's Note

All claims expressed in this article are solely those of the authors and do not necessarily represent those of their affiliated organizations, or those of the publisher, the editors and the reviewers. Any product that may be evaluated in this article, or claim that may be made by its manufacturer, is not guaranteed or endorsed by the publisher.
